# Burnout and coping strategies among resident physicians at an Indonesian tertiary referral hospital during COVID-19 pandemic

**DOI:** 10.1371/journal.pone.0280313

**Published:** 2023-01-20

**Authors:** Sri Linuwih Menaldi, Natalia Widiasih Raharjanti, Mardiastuti Wahid, Adhitya Sigit Ramadianto, Nadia Rahmadiani Nugrahadi, G. M. Yudi Prasetia Adhiguna, Dewi Anggraeni Kusumoningrum

**Affiliations:** 1 Department of Dermatovenereology, Faculty of Medicine Universitas Indonesia—Dr. Cipto Mangunkusumo Hospital, Jakarta, Indonesia; 2 Department of Medical Education, Faculty of Medicine Universitas Indonesia—Dr. Cipto Mangunkusumo Hospital, Jakarta, Indonesia; 3 Department of Psychiatry, Faculty of Medicine Universitas Indonesia—Dr. Cipto Mangunkusumo Hospital, Jakarta, Indonesia; 4 Department of Clinical Microbiology, Faculty of Medicine Universitas Indonesia—Dr. Cipto Mangunkusumo Hospital, Jakarta, Indonesia; Medical University of Vienna, AUSTRIA

## Abstract

**Background:**

The COVID-19 pandemic has increased the burden on resident physicians. They may use different coping strategies to manage those burdens, which partly determine their mental health outcomes, including burnout syndrome. This study explores the relationship between coping strategies and burnout among resident physicians during the COVID-19 pandemic in an Indonesian tertiary referral hospital.

**Methods:**

This online cross-sectional study was conducted from June to August 2020, involving nine residency programs in the Faculty of Medicine Universitas Indonesia–Cipto Mangunkusumo Hospital. Burnout syndrome was assessed using Maslach Burnout Inventory, while Brief COPE measured coping strategies.

**Results:**

A total of 388 residents participated in this study. High emotional exhaustion (EE), depersonalization (DP), and low personal accomplishment (PA) were found in 15.5%, 5.2%, and 39.2%, respectively. Residents more often use adaptive than maladaptive coping strategies. Higher PA was correlated to residents using problem-focused (r = 0.299; p < 0.001) and emotion-focused (r = 0.397; p < 0.001). Meanwhile, dysfunctional coping strategies are moderately correlated with EE (r = 0,518; p <0,001) and DP (r = 0,507; p<0,001).

**Conclusion:**

The use of dysfunctional coping strategies is linked to higher emotional exhaustion and depersonalization aspect of burnout. However, a higher sense of personal accomplishment is linked to problem-focused and emotion-focused strategies. Appropriate identification and intervention of residents with dysfunctional coping strategies may be beneficial in reducing burnout risk.

## Introduction

Medical residency is a period of significant emotional and physical stress, making it one of the most demanding stages in medical education [1, 2]. Stressors during residency may accumulate from the academic and clinical workload, workplace environment and organization, and difficulties in managing work-life balance [3]. Consequently, residents often face mental health issues such as depression, anxiety, and burnout syndrome. Among those issues, burnout is one of the most frequently reported and studied in resident physicians. It has been defined as a psychological syndrome characterized by emotional exhaustion, depersonalization, and low feelings of personal accomplishment [4].

Residents make up the majority of medical personnel in many academic hospitals, and the COVID-19 pandemic has been a source of additional stressors. Residents face a higher risk of COVID-19 infection and fear that they may transmit it to others, including vulnerable family members. They also face drastic changes at the hospital, such as working longer hours or more frequent shifts to cover for other residents who tested positive for COVID-19 or had to self-isolate due to exposure. Physical distancing and protective equipment requirements may impede communication with patients and colleagues [5, 6]. Beyond hospital walls, residents also have to manage pandemic stressors that affect the general population, such as mobility restrictions, social isolation, economic loss, and grief. As a result, residents have reported an increased burnout rate during the pandemic [5, 7].

Burnout negatively impacts residents’ psychological well-being, the quality of care they provide, and patient safety. Residents experiencing burnout are at risk of committing medical errors and neglecting proper infection control measures [5]. Furthermore, burnout is associated with other mental health issues such as depression, anxiety, and suicidal ideation.

Burnout results from the complex interaction between the workplace stressors mentioned above and the personal characteristics of the resident. A systematic review found that residents of younger age, female gender, unmarried status, certain specialties, and low level of job satisfaction are more likely to experience burnout [8]. Coping strategies the residents use to manage stress can also influence their risk of experiencing burnout [9]. Coping strategies are cognitive and behavioral efforts to modulate demands surpassing available resources [10, 11]. Some strategies may target the problem’s source, while others modify the emotional reaction to the problem. Additionally, some coping strategies are deemed dysfunctional. An Indonesian study found that dysfunctional coping correlates with the cynicism and emotional exhaustion experienced in burnout [12].

Although the impact of burnout is significant and wide-ranging, data on resident burnout in Indonesia is scarce among the 15 universities conducting residency training. The COVID-19 pandemic also added to the sense of urgency in this matter. This study explores the relationship between coping strategy and burnout among Indonesian resident physicians, particularly in the Faculty of Medicine Universitas Indonesia–Cipto Mangunkusumo Hospital, the national referral hospital in Indonesia. The results of this study may serve as a foundation for designing mental health interventions, especially those aimed at improving residents’ coping strategies. This study would serve well beyond the pandemic or other public health challenges.

## Methods

### Context

This study is a part of a more extensive study on burnout among physicians enrolled in residency training programs at the Faculty of Medicine Universitas Indonesia (FKUI) during the COVID-19 pandemic era. Residencies in Indonesia are university-based, and residents are considered postgraduate students placed at university academic hospitals or other teaching hospitals. In effect, they pay tuition each semester and do not receive a regular salary, although they may get financial incentives from hospital placements.

### Study design

This cross-sectional online study was conducted from June to August 2020, involving nine residency programs in FKUI. The residency programs were selected to represent different levels of COVID-19 exposure, with the high-level exposure group consisting of 5 programs: Pulmonology and Respiratory Medicine, Internal Medicine, Otorhinolaryngology, Anesthesiology, and Clinical Microbiology. The four programs considered low-level exposure groups consist of Dermatovenereology, Psychiatry, Obstetrics/Gynecology, and Anatomical Pathology. The programs are based in Cipto Mangunkusumo Hospital, except for Pulmonology and Respiratory Medicine, which is based in Persahabatan Hospital. All residents in the programs mentioned above were invited to participate in the electronic study channels. This study has been given ethical approval 554/UN2.F1/ETIK/PPM.00.02/2020 by the Health Research Ethics Committee of FKUI.

The research questionnaire consists of socio-demographic information and measurement of burnout and coping strategies. Collected socio-demographic data include gender, specialty, and stage of residency.

Burnout was assessed using the Maslach Burnout Inventory, a self-report instrument measuring three dimensions of burnout: emotional exhaustion (EE, nine items), depersonalization (DP, five items), and personal accomplishment (PA, eight items). The instrument has 22 items; each scored from 0 to 6. Results are analyzed for each domain; no composite score is used. EE score of 27 or more is categorized as high, 19 to 26 as moderate, and 18 or less as low. For DP, a score of 10 or more is considered high, 6 to 9 moderate, and 5 or less low. The scoring for PA is reversed: a score of less than 34 is considered high burnout, 34 to 39 is moderate, and 40 or more is low. The instrument has been adapted into Bahasa Indonesia with good validity and reliability [13].

Brief COPE measured residents’ coping strategies to manage stressors during the COVID-19 pandemic. It is a self-report instrument that covers 14 subscales representing different coping strategies. Each subscale consists of 2 items, scored from 1 to 4, which are then summed. A higher score on a subscale means that the respondent uses that specific coping strategy more frequently. The coping mechanisms in Brief COPE may also be grouped into problem-focused coping (active coping, planning, and use of instrumental support), emotion-focused coping (use of emotional support, positive reframing, acceptance, religion, and humor), and dysfunctional coping (venting, denial, substance use, behavioral disengagement, self-distraction, and self-blame) [14].

### Data analysis

Study data were managed and analyzed using SPSS version 25. Descriptive statistics are provided for demographic data and the prevalence of burnout. The relationship between coping mechanisms with gender and level of exposure was calculated using the Mann-Whitney test, while the relationship with the residency stage was tested using the Kruskal-Wallis correlation test. The correlation between coping mechanisms and burnout symptoms was tested using Spearman’s correlation test.

## Results

The online questionnaire was distributed to 524 residents from the nine selected programs. At the end of the study period, 388 residents had participated (response rate = 74%). Characteristics of study respondents are presented in Table 1. A majority of residents had low emotional exhaustion (70,1%) and depersonalization (85,3%), with more than a third of residents having low burnout as measured by personal accomplishment (36,3%). However, the level of EE is high in 15,5% of residents and moderate in 14,4%. DP is less frequent: 5,2% is classified as high and 9,5% as moderate. Residents are more evenly split between different levels of PA. As measured by PA, moderate burnout is found in 24,5% of residents and high in 39,2%.

**Table 1:** Demographic data of respondents (n = 388 participants).

Respondents (n = 388)		
Gender	Female	257 (64.7%)
	Male	131 (33.0%)
Level of exposure	Low	221 (55.7%)
	High	167 (42.1%)
Stage of residency	Junior	155 (39.0%)
	Intermediate	120 (30.2%)
	Senior	113 (28.5%)
Residency program	Anaesthesiology and Intensive Care	42 (10.6%)
	Dermatovenereology	52 (13.1%)
	Internal Medicine	12 (3.0%)
	Microbiology	18 (4.5%)
	Obstetrics and Gynecology	96 (24.2%)
	Anatomical Pathology	28 (7.1%)
	Psychiatry	45 (11.3%)
	Pulmonology and Respiratory Medicine	58 (14.6%)

This study’s respondents showed various coping strategies (Table 2). Scores for problem-focused and emotion-focused strategies are generally higher than dysfunctional coping. Among dysfunctional coping, the most frequently used are self-distraction, venting, and self-blame. Male residents tend to cope with humor and planning, whereas female residents are more likely to cope through venting and religion.

**Table 2:** Correlation between coping mechanism and gender, level of exposure, and stage of residency.

Coping Mechanism	Median (IQR)	Gender			Level of Exposure			Stage of Residency			
		Female^†^	Male^†^	*p-value* ^*‡*^	Low^†^	High^†^	*p-value* ^*‡*^	Junior^†^	Intermediate^†^	Senior^†^	*p-value* ^*§*^
**Problem-focused**	17 (15–19)	16(14–19)	17(15–18)	*0*,*532*	**17(15–19)**	**16(14–18)**	***0*,*003*** *	17(14–19)	17(15–19)	17(15–18)	*0*,*816*
Active coping	5 (5–6)	5 (4–6)	5 (5–6)	*0*,*564*	**6 (5–6)**	**5 (4–6)**	***0*,*018*** *	5 (4–6)	5 (5–6)	5 (5–6)	*0*,*870*
Planning	5 (5–6)	**6 (5–6)**	**6 (5–7)**	***0*,*049*** *	**6 (5–7)**	**6 (5–6)**	***0*,*006*** *	6 (5–7)	6 (5–6)	6 (5–7)	*0*,*567*
Instrumental support	6 (4–6)	6 (4–6)	5 (4–6)	*0*,*260*	6 (5–6)	5 (4–6)	*0*,*200*	6 (4–6)	6 (5–6)	5 (4–6)	*0*,*266*
**Emotion-focused**	28 (26–31)	29(26–31)	28(26–31)	*0*.*564*	29(26–31,5)	28(25–31)	*0*,*125*	28(25–32)	28(25–31)	29(26–31)	*0*,*419*
Acceptance	6 (5–7)	6 (5–7)	6 (5–7)	*0*,*669*	6 (5,5–7)	6 (5–7)	*0*,*488*	6 (5–7)	6 (5–7)	6 (5–7)	*0*,*609*
Humor	4 (3–5)	**4 (3–5)**	**4 (4–5)**	***0*,*032*** *	**4 (4–5)**	**4 (3–5)**	***0*,*000*** *	4 (3–5)	4 (3–5)	4 (3–5)	*0*,*730*
Religion	6 (5–7)	**6 (5–7)**	**6 (4–7)**	***0*,*007*** *	6 (5–7)	6 (5–7)	*0*,*540*	6 (5–7)	6 (5–7)	6 (5–7)	*0*,*605*
Emotional support	6 (5–7)	6 (5–7)	6 (5–7)	*0*,*353*	6 (5–7)	6 (5–7)	*0*,*650*	6 (5–7)	6 (5–7)	6 (5–7)	*0*,*568*
Positive reframing	6 (5–7)	6 (5–7)	6 (5–7)	*0*,*965*	6 (5–7)	6 (5–7)	*0*,*522*	6 (5–7)	6 (5–6)	6 (5–7)	*0*,*634*
**Dysfunctional **	22 (19–25)	**23(20–25,5)**	**21(19–24)**	***0*,*009*** *	**23(20–26)**	**21(19–23)**	***<0*,*001*** *	22(19–25)	22(19–25)	22(20–25)	*0*,*570*
Self-distraction	5 (4–6)	5 (4–6)	5 (4–6)	*0*,*372*	**6 (5–6)**	**5 (4–6)**	***0*,*000*** *	5 (4–6)	5,5 (4,25–6)	6 (4,5–6)	*0*,*147*
Denial	3 (2–4)	3 (2–4)	2 (2–3)	*0*,*157*	**3 (2–4)**	**2 (2–3)**	***0*,*001*** *	3 (2–4)	3 (2–3)	2 (2–4)	*0*,*963*
Venting	5 (4–6)	**5 (4–6)**	**4 (4–5)**	***0*,*002*** *	**5 (4–6)**	**4 (4–5)**	***0*,*004*** *	5 (4–6)	5 (4–5,75)	5 (4–6)	*0*,*656*
Substance use	2 (2–2)	2 (2–2)	2 (2–2)	*0*,*621*	**2 (2–2)**	**2 (2–2)**	***0*,*012*** *	**2 (2–2)**	**2 (2–2)**	**2 (2–2)**	***0*,*021*** *
Behavioral disengagement	2 (2–4)	2 (2–4)	2 (2–3)	*0*,*102*	**2 (2–4)**	**2 (2–3)**	***0*,*044*** *	2 (2–4)	2 (2–3)	2 (2–4)	*0*,*263*
Self-blame	4 (4–5)	4 (4–5)	4 (4–5)	*0*,*137*	**4 (4–5)**	**4 (3–5)**	***0*,*004*** *	4 (4–5)	4 (4–5)	4 (4–5)	*0*,*907*

^**†**^ Shown as median (Q1-Q3)

^***‡***^ Calculated using Mann-Whitney correlation test

^***§***^ Calculated using Kruskal Wallis correlation test

(*) statistically significant values (p-value <0,05) indicates significant differences within the group

Residents in the low exposure group showed higher scores for all types of dysfunctional coping: self-distraction (p = 0.000), venting (p = 0.004), denial (p = 0.001), substance use (p = 0.012), behavioral disengagement (p = 0.044), and self-blame (p = 0.004). On the other hand, residents in the high exposure group had lower scores for several problem-focused and emotion-focused coping: active coping (p = 0.018), planning (p = 0.006), and humor (p = 0.000). Meanwhile, senior residents scored higher in substance use than intermediate (p = 0.016) and junior (p = 0.034) residents.

Both problem-focused and emotion-focused strategies are correlated with higher PA (r = 0.299; p < 0.001 and r = 0.397; p < 0.001, respectively) (Table 3). The specific coping strategies that show moderate correlations with PA are planning (r = 0,313; p<0,001) and positive reframing (r = 0,474; p<0,001). Dysfunctional coping strategies are moderately correlated with both EE (r = 0,518; p <0,001) and DP (r = 0,507; p<0,001). This group finds the highest correlations for behavioral disengagement, self-distraction, and self-blame. There is also an inverse correlation between behavioral disengagement and personal accomplishment (r = -0,376; p<0,001).

**Table 3:** Correlation between coping mechanism and burnout symptoms.

Coping mechanism (2–8)		EE		DP		PA	
		*r*	*p value*	*r*	*p value*	*r*	*p value*
Problem-focused (6–24)		0,170	**0,001**	0,093	0,066	0,299	**<0,001**
	Active coping	0,152	**0,003**	0,073	0,153	0,243	**<0,001**
	Planning	0,145	**0,004**	0,066	0,196	**0,313** *	**<0,001**
	Instrumental support	0,105	**0,039**	0,109	**0,032**	0,185	**<0,001**
Emotion-focused (10–40)		-0,047	0,359	-0,014	0,776	**0,397** *	**<0,001**
	Acceptance	0,031	0,536	0,01	0,848	0,256	**<0,001**
	Humor	0,189	**<0,001**	0,283	**<0,001**	0,063	0,219
	Religion	-0,11	**0,03**	-0,143	**0,005**	0,235	**<0,001**
	Emotional support	-0,043	0,394	0,027	0,595	0,266	**<0,001**
	Positive reframing	-0,201	**<0,001**	-0,208	**<0,001**	**0,474** *	**<0,001**
Dysfunctional (12–48)		**0,518** *	**<0,001**	**0,507** *	**<0,001**	-0,192	**<0,001**
	Self-distraction	**0,368** *	**<0,001**	**0,331** *	**<0,001**	-0,044	0,392
	Denial	0,278	**<0,001**	0,261	**<0,001**	-0,179	**<0,001**
	Venting	0,201	**<0,001**	0,254	**<0,001**	0,014	0,784
	Substance use	0,178	**<0,001**	0,174	**0,001**	-0,067	0,187
	Behavioural disengagement	**0,454** *	**<0,001**	**0,46** *	**<0,001**	**-0,376** *	**<0,001**
	Self-blame	**0,38** *	**<0,001**	**0,385** *	**<0,001**	-0,09	0,076

Calculated using Spearman’s correlation; statistically significant values are bold (p-value<0.05)

(*) r 0.3 or r -0.3

## Discussion

Worldwide, there has been an increase in burnout among healthcare personnel after the COVID-19 pandemic. In Saudi Arabia, 27.3% of residents experience burnout and depression, with 26.4% having high emotional exhaustion, 10.7% high depersonalization, and 24.0% low personal accomplishment [7]. A study from Romania revealed that the average burnout for medical students was 76%, a higher percentage than in studies conducted before the pandemic [5]. Given its nature as a mechanism in response to stressors, different coping strategies can influence the risk of experiencing burnout [9]. In this study, we report coping strategies used by residents from different programs in the Faculty of Medicine Universitas Indonesia and how they relate to dimensions of burnout. This study is one of the country’s first studies on resident physician burnout. Although only 9 out of the 31 residency programs in FKUI were involved, the response rate among selected programs was relatively high, especially considering that online surveys tend to have lower response rates [15].

Residents use a variety of coping strategies. Problem-focused and emotion-focused coping is more frequently utilized than dysfunctional coping. There are also variations among the dysfunctional coping group, with venting and self-distraction having the highest scores. These results align with previous studies on coping strategies utilized by residents and medical students. Before the pandemic, a study on medical students in FKUI revealed similar coping patterns, with high emotion-focused and problem-focused coping scores. Self-distraction is also one of the most frequently dysfunctional coping mechanisms among students [12].

Studies of residents from various countries conducted before and during the pandemic concur with these results [16–18]. Although specific coping strategies differ, residents generally employ adaptive coping strategies more often than maladaptive ones. Respondents of this study rarely cope with substance use, which is a welcome finding in contrast to some studies that found a considerable prevalence of substance use among undergraduate and graduate medical students [19, 20]. Coping through religious practices is quite common in this study which may be attributed to local cultures and values [16, 21].

In previous studies using more open-ended questions on coping, residents and other healthcare workers also mentioned more practical ways to cope with pandemic stressors, such as increasing physical activity or exercise, getting enough sleep, ensuring adequate nutrition, and avoiding crowded places [17, 22, 23]. These strategies show that residents’ coping strategy is context-specific, partly shaped by the nature of the stressor itself, as fear of contracting COVID-19 and transmitting it to others is one of the main concerns among healthcare workers.

Findings that indicate residents employ many problem-focused and emotion-focused coping strategies point to the importance of coping flexibility, or the ability to adjust their coping according to specific demands of stressors. This study shows that residents have a rich repertoire of coping strategies, which suggests that they can use different strategies in different situations. Nevertheless, how they change their coping (coping variability) and suit it to current demands (coping fitness) needs to be explored further. Coping flexibility requires that individuals have the awareness to evaluate the effectiveness of their coping strategy, abandon it if needed, and formulate a more suitable strategy [24].

Our study found that, statistically, male residents are more likely to use humor and planning, while female residents cope with venting and religion. However, the actual Brief COPE score difference is slight, except for venting. Such gender differences can be found in some studies, although no consistent pattern can be discerned, while other studies found no difference [16]. In one study, female college students are more likely to use emotion-focused coping, mainly venting and getting emotional and instrumental support. It is suggested that female students have wider social networks that allow them to get more support [25]. Conversely, male students tend to detach from and not express their emotions outwardly. While focusing on problem-solving can be beneficial, repressing emotions can lead to other mental health issues. The lack of stark coping differences between genders may also point to more relaxed societal expectations regarding gender expression, as an endorsement of traditional masculinity and femininity correlates with problem-focused and emotion-focused coping [26]. Moreover, neurobiological factors cannot be completely discounted as evidence suggests male and female individuals respond differently to stress, such as in the hypothalamic-pituitary-adrenal axis and autonomic nervous system activation [27].

The different coping strategies between low and high-exposure groups in this study contrasted with previous studies. Frontline healthcare workers are more likely to use emotion-focused coping compared to non-frontliners [28]. In settings where the problem is not entirely understood, and the solutions are uncertain, problem-focused coping that aims to tackle the root(s) of the problem may not be effective. On the other hand, individuals can use emotion-focused coping to adjust their emotional reaction to the stressor, even though it has not been modified yet. Connectedness is an essential feature in emotion-focused coping. Studies on COVID-19 frontliners found that getting support from work colleagues, friends, and family is a preferred way to cope [29–31].

Residents coping strategies may also be influenced by more practical concerns. In the peak of COVID-19 hospitalizations, most technical and organizational support was directed toward residents working in high-risk areas. Thus, these residents are more prepared to care for COVID-19 patients. On the other hand, residents in low-risk areas might face more uncertainty as they could still be infected through patients who were not detected early enough. They also do not get the same level of support and resources compared to high-risk areas [16]. This contrast may explain why non-frontliners have higher scores for dysfunctional coping.

In this study, there are no significant differences in the coping mechanisms used by junior, intermediate, and senior residents. In contrast, previous studies show that patterns of coping strategy may change as residents progress through their residency. A nationwide study of residents from all specialties in Qatar found that junior residents employ avoidant coping styles more often than senior residents. Similarly, it was expected that FKUI residents would show a different coping pattern in each stage as each has its characteristics. For example, junior residents are still adapting to the residency program, gaining essential knowledge and skills in their chosen specialty, and practicing under close supervision of attendings and senior residents. As they become intermediate and senior residents, they have an immense workload with more complicated cases. They also have less supervision and are expected to transition into independent practice. Additionally, they may be responsible for the performance of their juniors. Nevertheless, a study of residents in Singapore also found similar coping responses between junior and senior residents, which may be attributed to the fact that this pandemic is an unprecedented situation for residents, regardless of their stage [32].

Residents who tend to use dysfunctional coping–specifically behavioral disengagement, self-blame, and self-distraction–also show higher scores of emotional exhaustion and depersonalization. On the other hand, personal accomplishment is positively correlated with planning and positive reframing. Additionally, higher behavioral disengagement is correlated with lower personal accomplishment. These findings align with existing literature from all levels of medical education in many parts of the world [18, 33, 34]. Burnout is more strongly correlated with various dysfunctional coping strategies, while its association with problem- and emotion-focused coping strategies tend to be insignificant.

These correlations support the notion that maladaptive coping strategies increase residents’ risk of burnout. However, it cannot be conclusively confirmed due to the cross-sectional design of this study. These coping strategies may be a form of reaction to the experience of being burnt out or are themselves symptoms of burnout. Moreover, some coping strategies labeled as “dysfunctional” may serve beneficial functions in certain contexts. For example, self-distraction and venting may provide immediate relief from psychological distress. Humans have limited working memory and attention capacity. When demands accumulate, individuals may need to cognitively disengage momentarily to adjust their priorities and formulate a more appropriate plan [25]. These strategies would be “dysfunctional” if the self-distraction is prolonged, leading to avoidance of the problem, or when venting is not followed by more adaptive strategies or done through inappropriate channels.

Residents who use dysfunctional coping to manage residency demands are prone to experience burnout, which may lead to other mental health issues and impair academic and clinical performance. Therefore, medical educators, especially residency program directors, should implement interventions to help residents modify their coping strategy, moving from dysfunctional to problem-focused and emotion-focused coping. Such mental health programs have gained traction recently as awareness about medical student and resident mental health continues to grow. As with any healthcare program, mental health interventions for residents should center on the experience and needs of residents themselves. Resident-designed and -led wellness programs are proven acceptable and effective in reducing burnout [35].

While burnout has been discussed mainly through a psychosocial lens, it should be noted that COVID-19 infection is associated with increased incidence and worsening of neuropsychiatric conditions such as strokes, headaches, depression, and anxiety, which in itself has been linked to burnout [36–40]. Possible mechanisms of pathogenesis are still being explored, including direct injury, hypoxic injury, and immune dysregulation [36, 41]. Further research may delineate how these biological factors contribute to the incidence of burnout and what extent.

## Study limitations

The respondents of this study come from nine out of thirty-one residency programs found in a tertiary referral hospital. Thus, although the programs have been chosen to represent residents with low and high exposure to COVID-19, it would be favorable for further studies with respondents from more residency programs and centers in Indonesia.

## Conclusion

Aside from adding to the growing body of research regarding the relationship between coping mechanisms and burnout, this study provides a novel picture of these themes in residents of a low-middle-income country during a global pandemic. Residents in FKUI employ different strategies to cope with residency demands during the COVID-19 pandemic, mainly problem-focused and emotion-focused coping. There are coping strategy differences between male and female residents and those in programs with low-risk and high-risk COVID-19 exposure. Dysfunctional coping strategies are generally associated with higher emotional exhaustion and depersonalization dimensions of burnout, while problem-focused and emotion-focused correlate with a higher sense of personal accomplishment. Identifying residents with dysfunctional coping strategies may allow for earlier burnout detection. Residency directors should provide appropriate mental health interventions to help residents use less dysfunctional coping strategies to reduce the risk of burnout.

## Supporting information

S1 Data Minimal data set. (XLSX)
